# Reply to R.V. Patel et al

**DOI:** 10.1200/GO.23.00293

**Published:** 2023-10-19

**Authors:** Masanori Mori, Cheng-Pei Lin, Shao-Yi Cheng, Sang-Yeon Suh, Sayaka Takenouchi, Raymond Ng, Helen Chan, Sun-Hyun Kim, Ping-Jen Chen, Kwok Keung Yuen, Maiko Fujimori, Takashi Yamaguchi, Jun Hamano, Yoshiyuki Kizawa, Tatsuya Morita, Diah Martina

**Affiliations:** Masanori Mori, MD, Division of Palliative and Supportive Care, Seirei Mikatahara General Hospital, Hamamatsu, Japan; Cheng-Pei Lin, RN, MSc, PhD, Institute of Community Health Care, College of Nursing, National Yang Ming Chiao Tung University, Taipei, Taiwan, Cicely Saunders Institute of Palliative Care, Policy and Rehabilitation, Florence Nightingale Faculty of Nursing, Midwifery, and Palliative Care, King's College London, London, United Kingdom; Shao-Yi Cheng, MD, MSc, DrPH, Department of Family Medicine, College of Medicine and Hospital, National Taiwan University, Taipei, Taiwan; Sang-Yeon Suh, MD, PhD, Department of Family Medicine, Dongguk University Ilsan Hospital, Goyang, South Korea, Department of Medicine, Dongguk University Medical School, Seoul, South Korea; Sayaka Takenouchi, RN, PhD, MPH, Department of Nursing Ethics, Division of Human Health Sciences, Graduate School of Medicine, Kyoto University, Kyoto, Japan; Raymond Ng, MBBS, MMed, FAMS, Palliative and Supportive Care, Woodlands Health, Singapore; Helen Chan, RN, BSN, PhD, The Nethersole School of Nursing, Faculty of Medicine, The Chinese University of Hong Kong, Hong Kong; Sun-Hyun Kim, MD, PhD, Department of Family Medicine, School of Medicine, Catholic Kwandong University, International St Mary's Hospital, Incheon, South Korea; Ping-Jen Chen, MD, PhD, Department of Family Medicine and Division of Geriatrics and Gerontology, Kaohsiung Medical University Hospital and School of Medicine, Kaohsiung Medical University, Kaohsiung, Taiwan, Marie Curie Palliative Care Research Department, Division of Psychiatry, University College London, London, United Kingdom; Kwok Keung Yuen, MBChB, MSc, FRCR, FHKCR, FHKAM, Department of Clinical Oncology, Queen Mary Hospital, Hong Kong; Maiko Fujimori, PhD, Division of Supportive Care, Survivorship and Translational Research, National Cancer Center Institute for Cancer Control, Tokyo, Japan; Takashi Yamaguchi, MD, PhD, Department of Palliative Medicine, Kobe University Graduate School of Medicine, Kobe, Japan; Jun Hamano, MD, PhD and Yoshiyuki Kizawa, MD, PhD, Department of Palliative and Supportive Care, Faculty of Medicine, University of Tsukuba, Tsukuba, Japan; Tatsuya Morita, MD, Division of Palliative and Supportive Care, Seirei Mikatahara General Hospital, Hamamatsu, Japan; and Diah Martina, MD, PhD, Department of Medical Oncology, Erasmus MC Cancer Institute, University Medical Centre Rotterdam, Rotterdam, the Netherlands, Department of Public Health, Erasmus MC, University Medical Centre Rotterdam, Rotterdam, the Netherlands, Division of Psychosomatic and Palliative Medicine, Department of Internal Medicine, Faculty of Medicine Universitas Indonesia, Jakarta, Indonesia, Cipto Mangunkusumo National General Hospital, Jakarta, Indonesia

We extend our sincere gratitude for the profound insights and perspectives offered by Patel et al^1^ on our review article^2^ regarding the concept of karma and its potential implications for symptom management at the end of life (EOL). We agree that karma might show various manifestations among different religions. Particularly, we appreciate the authors' consideration that focusing solely on the meaning of suffering in the context of karma could inadvertently promote the idea that patients may choose to endure suffering rather than seeking effective symptom management. We concur that providing symptom relief is paramount, and we support the authors' assertion that emphasizes the positive aspects of karma as a coping strategy within the realm of serious illness.

The principle of karma dictates that every action, whether good or bad, carries its own consequences. Life's challenges, such as a cancer diagnosis and physical suffering, could be interpreted as outcomes of negative deeds committed toward others in the past.^3-5^ While there exist a belief that undergoing physical suffering toward EOL might ameliorate adverse karma and lead to an improved subsequent life, the concept of karma also facilitates patients' acceptance of their circumstances and enhances their ability to cope with their life situations better.^6^ For example, a qualitative study involving a Thai women with breast cancer uncovered the perception that their illness resulted from bad karma, motivating patients to believe that performing virtuous deeds in their present life would yield better circumstances in the present or next life.^3^ Similarly, a qualitative study involving terminally ill patients with cancer in India indicated that karma was employed as a coping mechanism in their lives, to gain acceptance of the things happening to them.^7^ Furthermore, a large mixed-method study encompassing patients with oral cancer and multiple symptom clusters in India also demonstrated that patients accepted their situations with resilience, with their acceptance being intertwined with their beliefs in fate.^5^ Unlike passive acceptance, these patients actively sought cancer treatments, continued to explore ways to live with symptoms, and persevered in fulfilling the requisites to lead their lives.^5^

Considering the pronounced cultural diversity in Asia, we concur with the authors on the critical importance of future research on effective communication within specific cultures and religious contexts, such as karma. This research should involve stakeholder engagement to enhance our comprehension of the unique understanding or experiences of patients. There are different sects of the same religion across Asia. For example, Buddhists in Taiwan might have various religious concepts from those in Thailand, Korea, or Japan. As a result, the interpretation of karma could have subtle differences. Additionally, there exist individual variations in understanding and applying karma. Thus, the perspective on karma can be modified by who, where, and how it is interpreted. In this regard, we commend the authors for introducing community-based participatory research (CBPR), an innovative framework rooted in social justice that fosters collaboration between community members and academics to gain deeper insights into health-related challenges faced by their population.^8^ We applaud the efforts of one of the authors (R.V.P.) in conducting a CBPR project aimed at understanding how culture affects the palliative care preferences of Hindu patients with cancer and caregivers in the United States.^9^ Such a collaborative approach ensures that research aligns with the community's lived experiences and values, ultimately leading to culturally concordant care.

In sum, the intricate values, beliefs, and experiences of patients with cancer underscore the necessity for the development of interdisciplinary or cross-sectoral collaborative support through patient and public involvement. Urgent attention is warranted to illuminate the experiences of underrepresented Asian populations across the world. Future research will facilitate health care professionals in gaining a deeper understanding of these populations. These endeavors will further aid health care professionals in cultivating open-minded communication while upholding mutual respect.
